# External auditory exostoses and hearing loss in the Shanidar 1 Neandertal

**DOI:** 10.1371/journal.pone.0186684

**Published:** 2017-10-20

**Authors:** Erik Trinkaus, Sébastien Villotte

**Affiliations:** 1 Department of Anthropology, Washington University, Saint Louis, Missouri, United States of America; 2 UMR5199 PACEA, Université de Bordeaux–CNRS, Bâtiment B8, Allée Geoffroy Saint Hilaire, Pessac cedex, France; University of Delaware, UNITED STATES

## Abstract

The Late Pleistocene Shanidar 1 older adult male Neandertal is known for the crushing fracture of his left orbit with a probable reduction in vision, the loss of his right forearm and hand, and evidence of an abnormal gait, as well as probable diffuse idiopathic skeletal hyperostosis. He also exhibits advanced external auditory exostoses in his left auditory meatus and larger ones with complete bridging across the porus in the right meatus (both Grade 3). These growths indicate at least unilateral conductive hearing (CHL) loss, a serious sensory deprivation for a Pleistocene hunter-gatherer. This condition joins the meatal atresia of the Middle Pleistocene Atapuerca-SH Cr.4 in providing evidence of survival with conductive hearing loss (and hence serious sensory deprivation) among these Pleistocene humans. The presence of CHL in these fossils thereby reinforces the paleobiological and archeological evidence for supporting social matrices among these Pleistocene foraging peoples.

## Introduction

Paleopathological assessments of Pleistocene human remains have increasingly identified a suite of substantial and/or systemic developmental and degenerative abnormalities among the remains, in addition to an abundance of minor traumatic and oral lesions [1–5]. In addition, several abnormalities have been identified which would have impaired the normal functioning of the individuals, especially in the context of mobile Pleistocene foraging populations [6–10]. These alterations have suggested that the levels of social support present among recent humans (beyond the mother-child dyad) were present since the Early Pleistocene [6,9–11]. These inferences have implications for the levels of social integration and complexity among these non-modern members of the genus *Homo*.

In these assessments and inferences, however, there has been little consideration of impairments of the basic senses, ones which would have limited the abilities of the individuals to effectively perceive and respond appropriately to their natural and social environments. The only possible examples are post-traumatic unilateral ocular alterations in the Shanidar 1 Neandertal [6] and reduced conductive hearing in the Middle Pleistocene Atapuerca-SH Cr.4 from auditory exostoses [12]. The presence and degree of development of external auditory exostoses in the Shanidar 1 cranium may provide an additional, and substantial, case of such sensory impairment among these Pleistocene hunting-gathering populations.

External auditory exostoses (EAE) are bony growths into the auditory canal from the tympanic and/or squamous walls of the external auditory meatus and the margins of the auditory porus [13,14]. They vary from small rounded protrusions to processes that largely fill the meatus, are usually limited to the lateral opening of the canal (the porus), but may extend medially to the area of the tympanic membrane. They normally do not involve or develop from the tympanosquamous or tympanomastoid sutures; such sutural protrusions are osteomata, or benign neoplasms, which normally occur laterally within the meatus, are less frequent and are often solitary [14,15] (S3 Text). Either one can reduce the lumen of the auditory meatus.

EAE have been extensively documented in clinical settings and Holocene skeletal samples (see reviews in [13,16,17]). Their etiology in individual cases is not always apparent, but in general they are associated with prolonged exposure of the auditory canal to cold water (for a review, see [16]). They occur in elevated frequencies among modern participants in cold water sports (hence references to “swimmer’s/surfer’s ear”) (e.g. [18–24]). EAE have also been extensively recorded in Holocene skeletal samples, with particular attention focused on samples of people frequently engaged in aquatic resource exploitation (e.g. [17,25–34]).

EAE presence has been noted in a couple of Middle Pleistocene humans [12,35], several Late Pleistocene archaic humans [36–39], and a few early modern humans [40], and their absence has been noted in a few other Pleistocene humans [41–43]. Yet, the only discussion of the effects of such changes in the auditory canals among these Pleistocene humans has been the mention of deafness in the Atapuerca-SH Cr.4 [12]. The pronounced ones of the Shanidar 1 Neandertal, however, may provide further insight into these aspects of Pleistocene humans.

## Materials and methods

**Fig 1:**
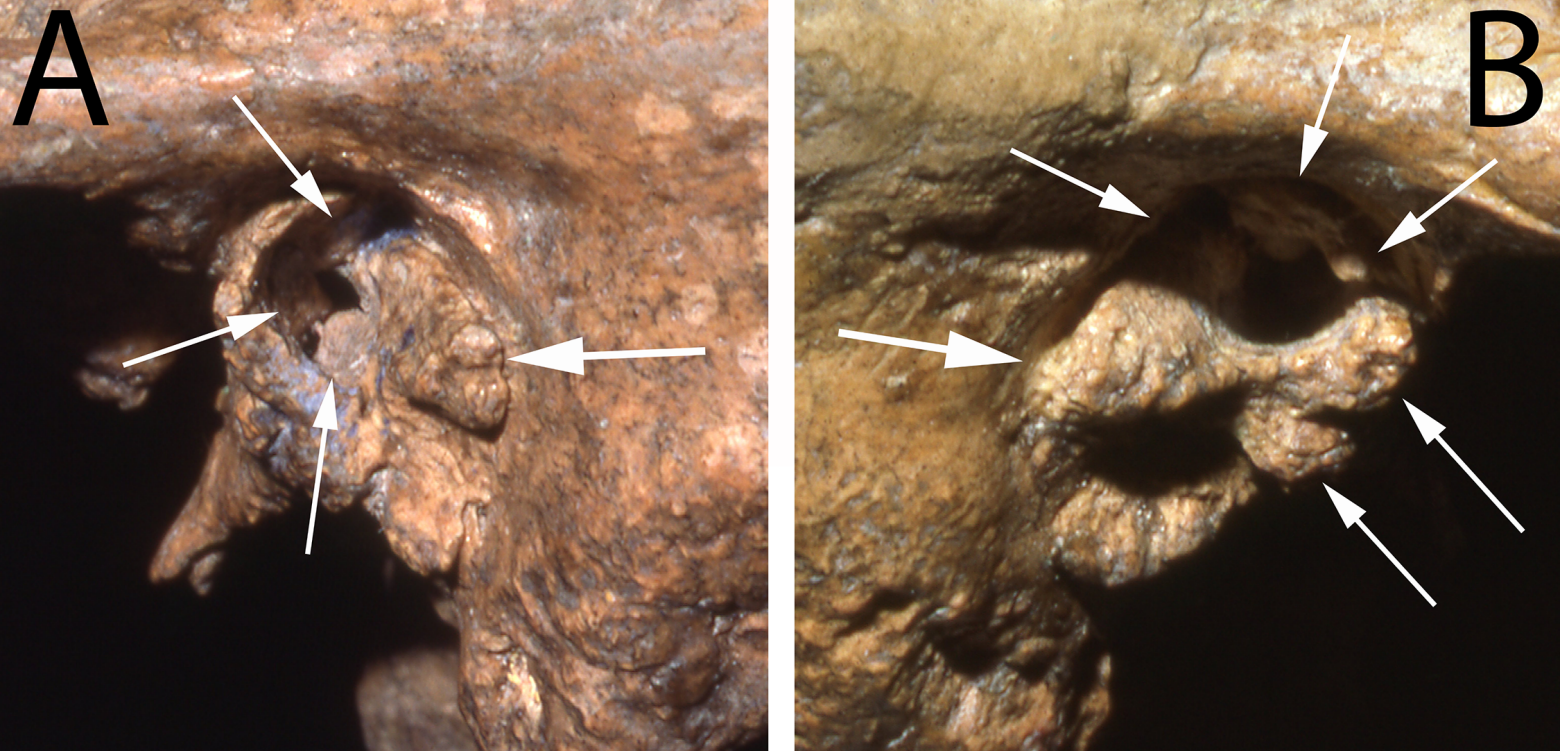
The temporal bones of Shanidar 1. A and B: lateral views of the left and right external auditory meatus illustrating the large external auditory exostoses, especially the bridging ones on the right side. The arrows point to the exostosis growths described in the text.

The ≈50 ka BP Shanidar 1 Neandertal cranium [6,44] (S1 Text) was analyzed visually with low magnification assessment of the intact right and left external auditory meatus in the Iraq Museum, Baghdad in 1976–78. It is part of the largely complete skeleton of an adult (40–50 years old, based on his pubic symphysis and dental wear comparisons to the histologically aged Shanidar 2 to 6) male (pelvically sexed based on the greater sciatic notch) [6,45]. Cranial radiography was not available in the Iraq Museum, and reanalysis since then has not been feasible. Observations are therefore based on the externally visible configurations of the auditory pori and lateral meatus (Fig 1).

The degree of development of the EAE is scored using an ordinal scale of Grade 0 (absence of EAE) to Grade 3 (large EAE that largely block the meatus) (Grade 1: <1/3; Grade 2: 1/3–2/3; Grade 3: >2/3) [19,25,26] (see also [13]). Similar observations are provided for four other Neandertals with EAE, the younger adult Spy 1 and Tabun 1 and the modestly older La Chapelle-aux-Saints 1 partial skeletons, plus the Krapina 39.1 isolated mature temporal bone (S2 Text).

## The Shanidar 1 external auditory meatus

Both of the external auditory meatus (EAM) of Shanidar 1 are well preserved; inferiorly the tympanic and adjacent portions of the temporal bone present little of note (Fig 1, S1 Text). The tympanic bones are complete, fully fused to the squamous and petrous portions, with complete closure of the foramina of Huschke.

The left porus (Fig 1A) has a normal anterior portion of the tympanic bone, that leads into a thick and inferiorly protruding tympanic bone against the mastoid process. There are no alterations of the adjacent squamous and mastoid bone. There is a large growth protruding laterally and anteriorly into the porus along all of the posterior side of the porus. It is on the lateral surface of the posterosuperior tympanic bone, but it extends anterosuperiorly into the porus and more medially into the meatus. Superiorly, this exostosis is very close to a small exostosis within the meatus, one which grew posteroinferiorly from the superior portion of the tympanic bone, immediately below the tympanosquamous suture. In addition, deeper within the meatus but laterally visible, there is a rounded protrusion extending close to the middle of the canal from the posteroinferior tympanic bone and a smaller one deep on the anterior tympanic bone. Together these growths provide the Shanidar 1 left EAM with a Grade 3 score. The lateral opening of the canal was reduced to a very small one in the middle of its anterior portion.

The right external auditory meatus and porus of Shanidar 1 (Fig 1B) has a more pronounced EAE development than the left one. The inferolateral margin of the tympanic bone is normal, as are the surrounding squamous and mastoid portions. Protruding laterally and especially anteriorly from the mid-posterior tympanic bone is a large and bulbous exostosis, the major portion of which extends to the middle of the meatus. Its posterior portion continues superiorly to where it approaches the superior extent of the anterior tympanic bone. But across the middle of the opening, the large exostosis narrows and then connects to a pair of more modest exostoses, which extend laterally and posteriorly from the mid-anterior tympanic bone.

This combination of right meatal growths completely blocks the middle of the porus, leaving only small openings anterosuperiorly and posteroinferiorly. They are then joined by a small protuberance within the meatus projecting inferiorly from the superior-most anterior tympanic bone. These large EAE, and especially the bridging of them across the middle of the porus, provide the Shanidar 1 right auditory canal with a serious Grade 3 score.

Data on the shapes of the more medial canal are not fully available; the visible EAE may extend medially along the auditory canals, as is indicated by the small ones within each meatus. The medial canals adjacent to the area of the tympanic membrane are nonetheless conservatively assumed to be normal.

The external auditory meatus of this moderately older Neandertal therefore present slightly different patterns of abnormalities. The left one approximates the marked patterns occasionally encountered in recent humans. The right one extends to the upper end of the four-part ordinal scale.

## Discussion

### Shanidar 1

The degree of development of the Shanidar 1 EAE is associated with conductive hearing loss (CHL) in extant humans [19,21,23,24,46–49]. Most EAE are located laterally in the auditory canal; as a result, usually they do not impinge directly on the tympanic membrane. Yet, they may extend medially, causing stenosis of the canal and associated CHL [50]. Even though individuals vary in their rates of production of cerumen and responses to irritation of the auditory canal, large (Grade 3) EAE would make it extremely difficult for the normal irrigation of the ear canal to cleanse the cerumen and exogenous debris from the canal [14,47,51]. The accumulated material, in combination with the exostoses, would then reduce both the sound transmission through the ear canal and the ability of the tympanic membrane to transmit the sound waves to the middle ear [52,53]. Impacted cerumen and exogenous material in the canal is therefore a common cause of CHL in recent humans.

The left meatus of Shanidar 1 is likely to have accumulated sufficient material to produce CHL, given its large posterior exostoses across at least half of the lateral opening. The right one, however, would have led to the presence of cerumen and other debris behind the bony bridging that connects the anterior and posterior exostoses. It would have been essentially impossible for Shanidar 1 to maintain a sufficiently clear canal for adequate sound transmission. He would therefore have been effectively deaf in his right ear, and he likely had at least partial CHL in the left ear.

Consequently, Shanidar 1 appears to have had an advanced degree of unilateral conductive hearing loss and reduced hearing acuity on both sides. In addition to a general reduction in hearing acuity, unilateral CHL limits one’s ability to discern the signal from background noise and to locate sounds in space. Among modern urban children it is associated with varying degrees of reduced academic progress [53], and hearing loss in living adults is associated with difficulties in communication, information exchange and social interactions, decreased mental and physical function, isolation, and psychological disorders [54–56].

In addition to a reduced effectiveness in communication and coordinated social activities (among these fully linguistic Paleolithic foragers [57–59]), it would have had more direct consequences. Hearing acuity is related to hunting effectiveness among recent human foragers [60]. Hearing is an important component of learning lithic technology [61]. Well developed auditory acuity is especially important in providing feedback during multistep lithic reduction that requires the fashioning of both sequential striking platforms and the desired tool [61]; this requirement applies to Acheulian bifaces and subsequent lithic procedures. CHL would also have made the individual vulnerable to medium to large carnivores, predators that were ubiquitous in Late Pleistocene Eurasia [62], the Zagros Mountains [63] and at Shanidar Cave [64].

Shanidar 1 also experienced a suite of other degenerative difficulties [6,44,65] (S1 Text). He is best known for his withered right shoulder and arm, that was little more than a weakened stump extending to just proximal of the elbow; it was most likely amputated above the elbow, possibly after a non-union fracture and associated atrophy. He had a laterally crushing fracture of the left orbit (probably altering and/or reducing vision). He experienced right genual and pedal trauma and osteoarthritis producing an abnormal gait (reflected in right talar remodeling and left tibiofibular posterior bowing), thereby impairing his landscape mobility and agility. And there is evidence of probable hyperostotic disease (DISH), which is associated with muscular tendinosis and reduced back and appendicular ranges of motion. It is therefore in addition to these degenerative conditions that any degree of hearing loss would have compounded the limited abilities of this individual to function.

### Other Pleistocene human external auditory exostoses

As noted above, external auditory exostoses have been observed in other Pleistocene humans, but most of them are modest in size. Four other Neandertals, La Chapelle-aux-Saints 1, Krapina 39.1, Spy 1 and Tabun 1, exhibit large EAE, ones which would be scored as Grade 2 [S2 Text]. Their bony growths are less likely by themselves to have produced advanced CHL, but they probably reduced the abilities of those individuals to cleanse their auditory canals and hence maintain auditory acuity.

In addition, the Middle Pleistocene (≈430 ka) Atapuerca–SH Cr.4 [66] developed bilateral Grade 2 EAE, but it resulted in atresia of the auditory canal [12]. This narrowing of the canal is usually a rare congenital condition, occurring in 1 out of 10,000–20,000 individuals, most often unilaterally [14,67,68]. The condition in Atapuerca-SH Cr.4 appears to be the result of the EAE extending medially and almost entirely blocking the canals. The degree of CHL associated with this degree of aural atresia would be moderate to severe [53], and it would therefore have had consequences similar to those for Shanidar 1.

### Pleistocene human social support

The advanced EAE of Shanidar 1, as well as that of Atapuerca-SH Cr.4, and the consequent CHL raise the question of social assistance among Pleistocene archaic *Homo*. The presence of social support among non-modern Pleistocene humans, as noted above, has been inferred for a number of Pleistocene individuals with substantial abnormalities and varying degrees of loss of function (e.g. [6,7,9–11,44] (see [69]). Comparisons with non-captive non-human primates, however, have questioned whether some of these abnormalities would have been sufficient to require social support in order for the individual to survive [70–72] (note, however, that the survival of macaques with congenital limb deformities documented by Turner et al. [72] is not relevant, because they were provisioned and hence had “social support”). For example, apes born with congenital disorders are sometimes maintained by their mothers for extended periods of time through infancy [73]. Apes lacking one eye appear to manage effectively in the wild [74]. Apes who have lost a hand or a foot (as in a snare) are able to climb and forage, although less effectively than their conspecifics [75,76]. And wild-shot primates are known (if rare) with up to two thirds of the dentition lost antemortem [10,77].

The non-human primate examples provided here are relevant for inferences regarding several Neandertals with partial loss of function. For example, the survival of the developmentally abnormal of the Pech-de-l’Azé 1 Neandertal child [78] should represent maternal support of an affected offspring. The apparent unilateral impaired vision of Shanidar 1, by itself, may not have limited his abilities. The upper limb fractures of Feldhofer 1 and Krapina 180 and 188.8 [79,80], as well as the right arm and shoulder of Shanidar 1, limited or severely reduced the utilities of the affected arms; yet, by themselves they would have implied only partial loss of foraging ability. The extensive antemortem tooth loss of La Chapelle-aux-Saints 1, and the less pronounced losses of Shanidar 4 and 5 [6,81], need not have limited their abilities to ingest food. However, the Aubesier 11 Neandertal and the Early Pleistocene Dmanisi D3444/D3900 experienced extensive antemortem tooth loss and (more importantly) pervasive infectious alteration of the maxilla and/or mandible [9,10]; they are joined by the Guattari 1 edentulous cranium with palatomaxillary inflammation [82]. Considerations of these fossils that only addressed the tooth loss [70,71] missed the point; these individuals had severely impaired oral tissues and not merely a loss of teeth. In addition, as emphasized by Hublin [69], the long term persistence of the Middle Pleistocene Atapuerca-SH Cr.14 and Salé 1 with substantial congenital abnormalities (cranial synostosis and torticollis, respectively) [7,11] imply care beyond the infantile maternal support evident among non-human primates.

In this context, the conductive hearing loss (CHL) of Shanidar 1 and Atapuerca-SH Cr.4 join several other Pleistocene archaic *Homo* individuals (if not all of the ones that have been invoked in the past) in indicating some level of social support. Moreover, although one could discuss the degrees to which support would have been necessary for the survival of those individuals with degenerating mastication or cranial developmental defects, an individual with advanced CHL would have been highly vulnerable alone in a Pleistocene foraging context (see above). For Shanidar 1, the CHL was associated with loss of function in other aspects of his biology, all of which would have compounded his need for support, even if some of the individual deficiencies by themselves would not have required such assistance.

The inferred presence of social support among at least the Neandertals should not be surprising. There is abundant evidence of intentional burial of the dead [83], even if not all of the known remains derive from such burials. Explicit mortuary practice reflects, ultimately, the presence of social cohesion, social roles, and hence mutual support [84,85], such as would have led to the caring of the impaired. There are items of personal decoration [86–88] and the use of pigments [89,90], which are modifications of one’s visual persona and hence a reflection of social identity manipulation and social cohesion. A number of Neandertal sites exhibit distinct spatial organization (e.g. [91–94]), reflecting coordinated use of the occupied space. There is evidence for division of labor by age and sex among them [95,96], reflecting the social integration of different roles. Moreover, whatever the Late Pleistocene human population dynamics might have been [97,98], it is increasingly apparent that the behavioral differences between the Neandertals and their modern human contemporaries and successors were modest [99–101].

## Conclusion

The Shanidar 1 Neandertal, in addition to his other traumatic and degenerative lesions, developed advanced external auditory exostoses, largely blocking the meatus on the left side and bridging across the auditory porus on the right side. Although a normal bony growth in the context of irritation of the external auditory canal, the extent of exostosis development in Shanidar 1 indicates a marked degree of conductive hearing loss. It joins the Middle Pleistocene Atapuerca-SH Cr.4, with its auditory atresia and associated deafness, in indicating survival of these sensorily impaired archaic humans despite the rigors and dangers of a Middle-to-Late Pleistocene foraging existence. A substantial degree of social support, especially given Shanidar 1’s plethora of other impairments, is indicated.

## Supporting information

S1 Text The Shanidar 1 abnormalities (PDF)

S2 Text Neandertal external auditory exostoses. (PDF)

S3 Text Differential diagnosis. (PDF)
