# Supplementary material for: External auditory exostoses and hearing loss in the Shanidar 1 Neandertal
Source: PLoS One. 2017 Oct 20;12(10):e0186684. doi: 10.1371/journal.pone.0186684 (PMC5650169; doi:10.1371/journal.pone.0186684)
Supplement: S1 Text — (PDF) [file pone.0186684.s001.pdf]

External Auditory Exostoses and Hearing Loss in the Shanidar 1 Neandertal Supporting Information Appendices

Erik Trinkaus[1] and Sébastien Villotte[2]

[a] Department of Anthropology, Washington University, Saint Louis MO 63130, USA
[b] UMR5199 PACEA, Université de Bordeaux – CNRS, Bâtiment B8, Allée Geoffroy Saint Hilaire, CS 50023, 33615 Pessac cedex, France

S1 Text: The Shanidar 1 abnormalities

During the restoration and analysis of the Shanidar 1 remains by T.D Stewart in 1957, he recognized that the individual had sustained multiple traumatic injuries [1-3]. In particular, the reduced form of the right humerus was noted by him, as were injuries to the cranium. Subsequent analysis of the Shanidar 1 remains by ET in 1976 and 1978 in the Iraq Museum further documented these traumatic injuries, noted additional abnormalities on the remains, and provided a description of the lesions [4]. These descriptions and assessments resulted in the formulation of alternative scenarios for the accumulation of these skeletal lesions [4,5], but the focus at that time was on the diagnosis of the lesions and the inferred elevated levels of risk of injury among the Shanidar and other Neandertals. Only passing reference was made to the disabling conditions of Shanidar and the probability of social care [4,6].

Subsequently, there have been further assessments of the right shoulder and arm abnormalities [7] and a diagnosis of hyperostotic disease (diffuse idiopathic skeletal hyperostosis (DISH), Forestier's disease) has been suggested [8] for the tendinous and ligamentous ossifications on the remains. The bilateral auditory exostoses were noted by Stewart [1] and ET [4]. However, they were considered to be of unknown etiology [1] and "not particularly unusual" [4]. In neither case were they assessed in terms of possible hearing loss, and hence no reference was made to their behavioral implications.

In this context, although the abnormalities of Shanidar 1 have been variably described and paleopathologically assessed, it is appropriate to summarize and illustrate them here to provide a framework in which to assess his auditory exostoses and their implications.

Shanidar 1 cranial trauma

Shanidar 1 (Fig S1), as with Shanidar 5 and a substantial number of Pleistocene human remains [9,10], sustained minor exocranial injuries to the neurocranial vault. The right frontal squamous presents several irregularities, first noted by Stewart [1], that represent minor trauma to the scalp and the underlying pericranium. They only affected the external table and are unlikely to have caused more than temporary discomfort.

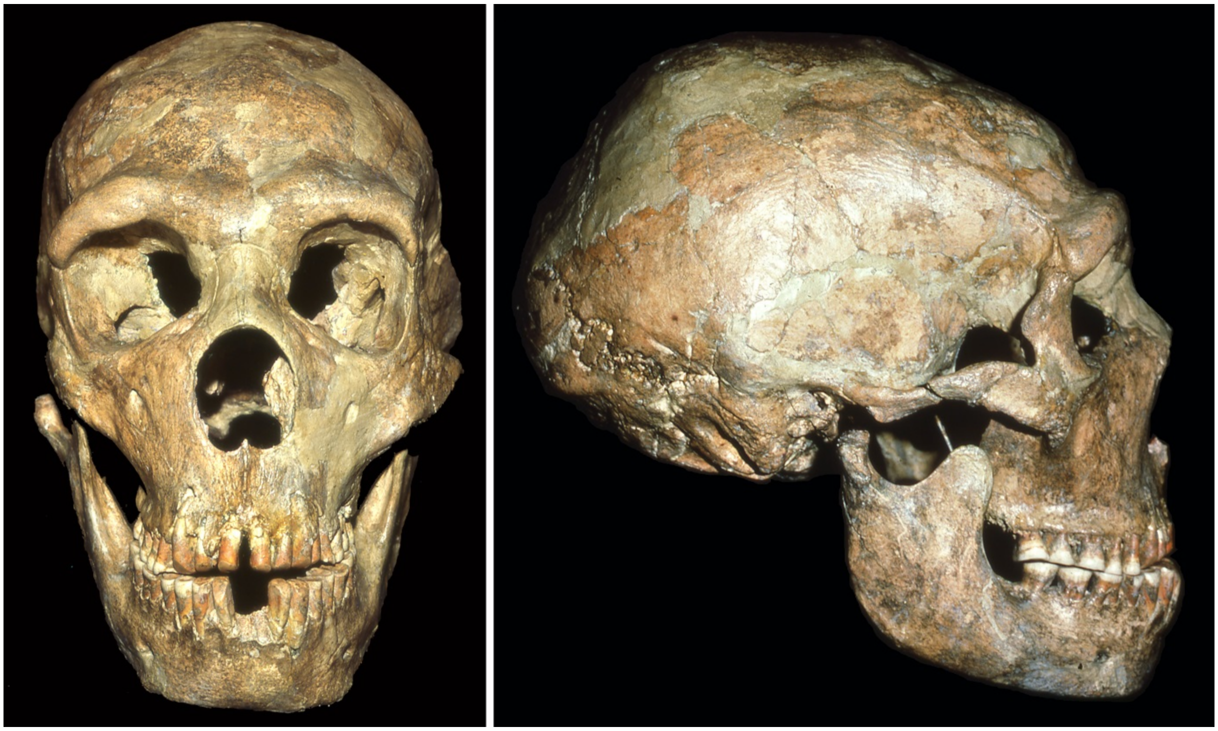

S1 Fig. Anterior and lateral views of the Shanidar 1 Neandertal skull. The surficial pericranial lesions are evident on the right frontal squamous portion.

However, he sustained a crushing fracture to the lateral left orbit (Fig S2). It fully healed, but resulted in an abbreviation of the lateral supraorbital torus, a flattening of the lateral orbital margin (primarily on the frontal process of the zygomatic bone), an anterolateral compression and irregularity of the zygomatic bone, and a reduction in the mediolateral breadth of the orbit (reduced 3.5 mm, or 7.3% of the right orbital breadth). It is unclear to what extent the orbital narrowing would have impinged (directly or through surrounding tissues) on the ocular sphere, but it is likely to have affected the position of the lateral rectus muscle. Shanidar 1 may therefore have had reduced left eye vision and/or ocular coordination as a result of the compressing fracture.

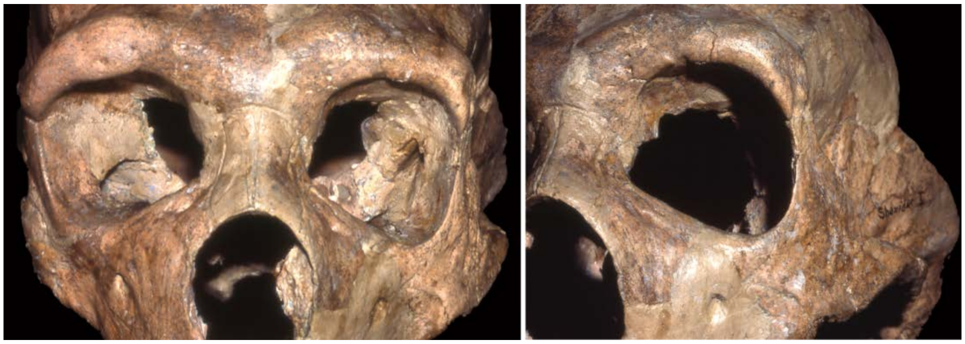

S2 Fig. The Shanidar 1 orbital region in anterior view (left) and an anterolateral view of the left lateral orbital region (right). The healed left crushing fracture extends from the lateral supraorbital torus to the inferior margin of the zygomatic body.

Shanidar 1 right humeral abnormalities

The left humerus of Shanidar 1 (Fig S3B) is normal, and it exhibits levels of musculoligamentous and diaphyseal cortical bone hypertrophy similar to those of other Shanidar and Neandertal humeri [4,11]. The right humerus, in contrast, is a narrow and angled diaphysis, albeit one with clear evidence of the bicipital sulcus, the pectoralis major tuberosity, the supracondylar crests, and the olecranon fossa (Figs S3A and S3C). The proximal two-thirds of the diaphysis are straight, but the diaphysis then angles mediodistally and ends at the level of the proximal olecranon fossa. The irregular angulation, with an S-curve in anterior view and a posterior displacement in medial view (Fig S4), is the product of at least two diaphyseal fractures, breaks which healed with distortion and disruption of the medullary cavity (Fig S4C). The diaphyseal cortical bone remains proportionately relatively thick throughout, with a mid-proximal percent cortical area of 89%. The distal end (Fig S4D) consists of exposed trabeculae, the margins of which are rounded over. There are irregular lacunae within the distal trabeculae (Fig S4C). The length of the right humerus from the surgical neck to the proximal olecranon fossa (243 mm) is ≈10% shorter than the same length estimated for the left humerus.

Differential diagnosis of the Shanidar 1 humerus [4,7] indicates that the primary incident producing the abnormalities was a set of fractures to the distal humerus. The fractures and subsequent healing produced the angular changes in the distal diaphysis. The distal end, with its irregular but rounded over trabeculae, should represent either the proximal side of a non-union fracture (pseudoarthrosis) or the end of an amputated arm; one side of a non-union fracture can resemble the amputation of a limb segment with a single diaphysis [12]. No trace of the more distal right arm or of the right hand was located *in situ* despite the preservation of anatomically neighboring elements [2]; amputation, probably subsequent to humeral atrophy with a pseudoarthrosis, is therefore indicated. It is unclear whether it would have been an auto-amputation or done with the assistance of another member of his social group; the former is possible for the removal of a withered forearm and hand at a pseudoarthrosis, whereas the latter would appear to have been necessary if done close to the time of the injury.

The maintenance of cortical thickness, despite the slender diameter of the diaphysis and loss of the subcubital limb, suggests some level of muscle tone along the humeral diaphysis. At the same time, the shortness of the bone relative to the estimated length of the left humerus, suggests that the injury occurred prior to full maturation, or some time during adolescence. Given the age-at-death of Shanidar 1 during the fifth decade (based on pubic symphyseal morphology and dental wear relative to the histologically aged other Shanidar Neandertals [4,13]), he would have lived with the humeral alterations for more than two decades.

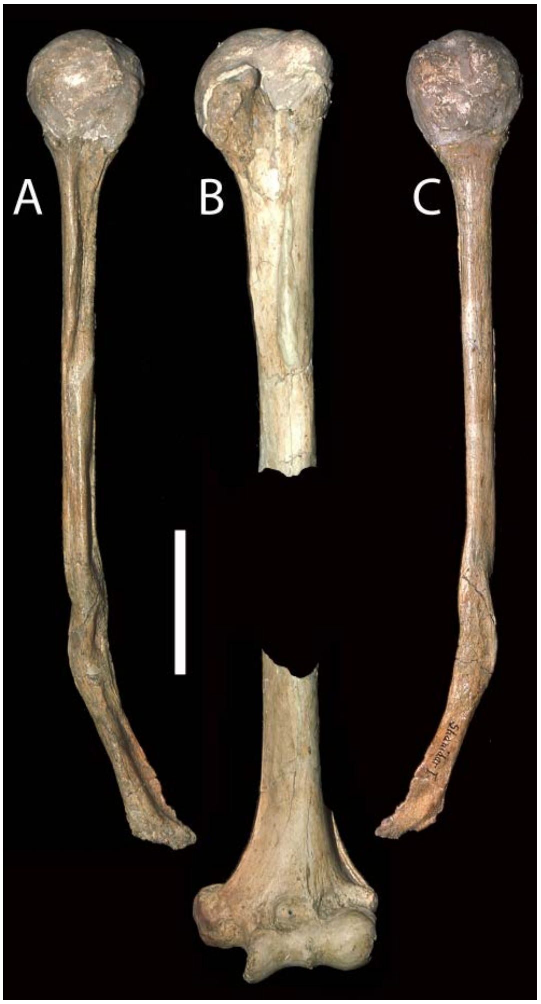

S3 Fig. The Shanidar 1 humeri. A and C: the abnormal right humerus in anterior (A) and posterior (C) views. B: the normal left humerus in anterior view. The head of the right humerus was modeled in plaster by T.D. Stewart and is approximate. The missing mid-distal diaphyseal section of the left humeral diaphysis was not preserved; the proximal and distal portions have been positioned approximately based on Late Pleistocene and recent human humerofemoral length proportions. Scale bar: 5 cm.

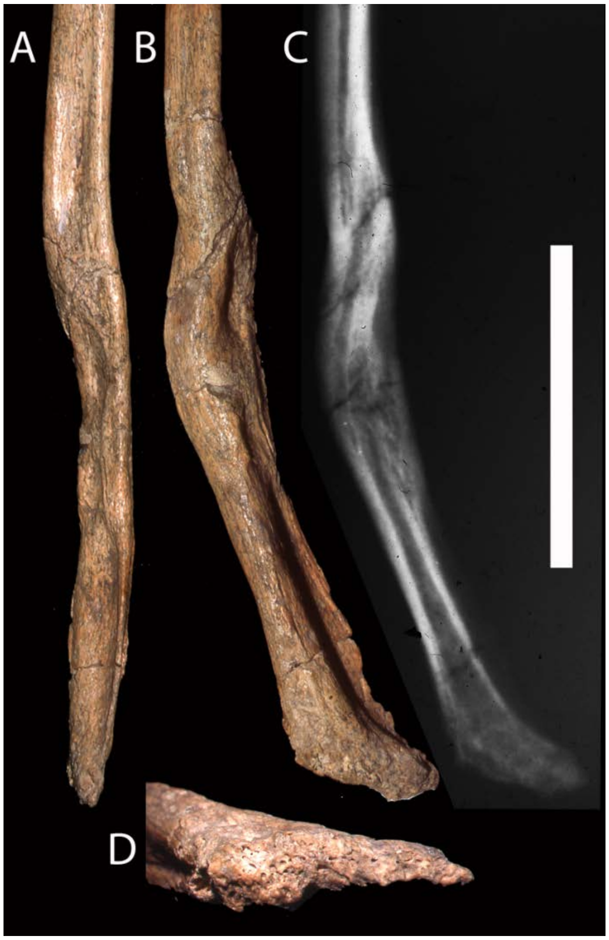

S4 Fig. The Shanidar 1 distal right humerus. A: medial view; B: anterior view; C: anteroposterior radiograph; D: distal view. Scale bar for A to C: 5 cm; D is enlarged.

Shanidar 1 shoulder abnormalities

In conjunction with the humeral fractures and its atrophy/hypotrophy, the right scapula and clavicle of Shanidar 1 are substantially smaller than the normal left ones. The right scapula (Fig S5) is incomplete, but its largely complete spine is 75% to 80% of the length of the complete left one. Its spine height is ≈65% of the height of the left scapular spine. The two bones, to the extent preserved, appear otherwise normal.

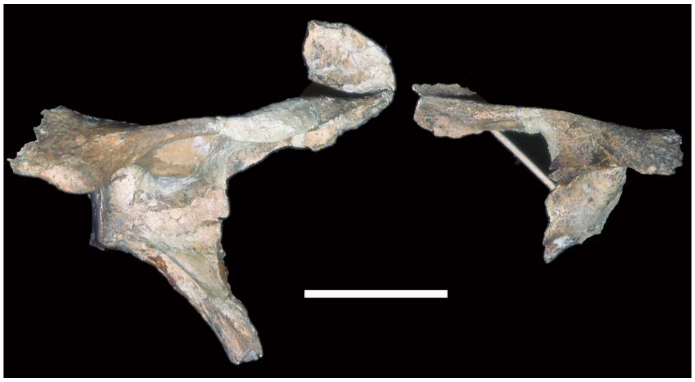

S5 Fig. **The Shanidar 1 scapulae in dorsal view**. The less complete right one preserves the spine from the base of the acromion process to close to the vertebral border, plus dorsal surface bone to a small infraglenoid portion of the axillary border. The left one extends from the middle of the acromion process to the vertebral border. Scale bar: 5 cm.

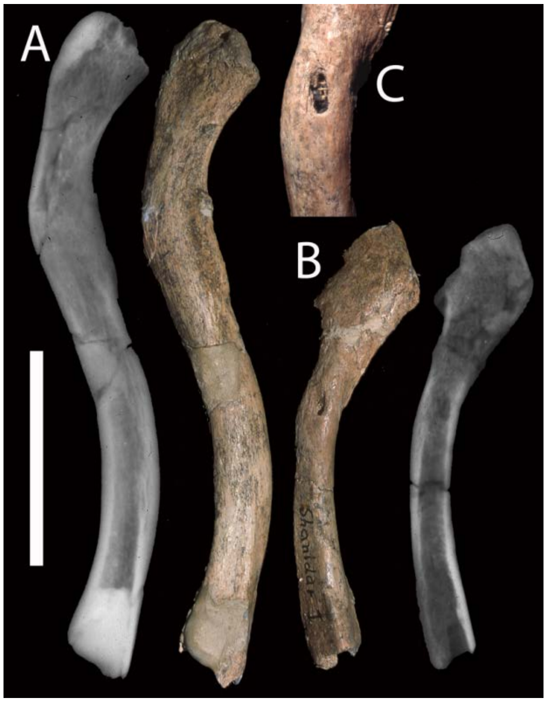

S6 Fig. **The Shanidar 1 clavicles**. A: superoinferior radiograph and superior view of the normal left clavicle. B: superior view and superoinferior radiograph of the right clavicle. C: enlarged detail of the osteomyelitis cloaca and involucrum on the right clavicle. Scale bar for superior views and radiographs: 5 cm.

The right clavicle is also considerably smaller than the left one in apparent length and in its shaft diameters (Fig S6). The smaller external diaphyseal diameters are also reflected in thinner cortical bone. These changes are accompanied in the left clavicle by a healed osteomyelitis lesion, with an open cloaca containing rounded trabeculae and an involucrum that spread across the cranial surface of the distal diaphysis (Fig S6C). This lesion, however, is the only sign of infection on the Shanidar 1 remains, despite the numerous lesions, and it healed long before death.

Shanidar 1 lower limb degenerative abnormalities

The Shanidar 1 lower limbs retain most of the right femur, most of the left tibia and fibula, both patellae, the right talocrural and tarsometatarsal skeletons, and the left medial tarsometatarsal skeleton. The only articular facets with subchondral bone degeneration are the right first tarsometatarsal ones, but there are periarticular changes in the right knee, ankle and hallux and remodeling of the right talus and the left tibia and fibula (Figs S7 and S8). There are additional enthesopathies on the patellae and calcanei probably related to DISH (see below; Fig S9).

The right lateral femoral condyle has pronounced periarticular exostoses extending close to the lateral epicondyle (Fig S7A). At the right talocrural articulation, there are ossifications of the flexor hallucis longus bursa and the posterior articular capsule on the distal tibia (Figs S7B and S7C), as well as exostoses around the anterior (and to a lesser extent posterior) talar trochlea and medial malleolar surface (Fig S7D). More pronounced is the general flattening of the right talar trochlea, evident in medial view (Fig S7E), which involved extensive remodeling of the talar trochlear region but not of the plantar (talocalcaneal) portions of the bone.

These talocrural changes are associated with an advanced degeneration of the first and second tarsometatarsal region, with periarticular bone deposition and resorption around the first tarsometatarsal articulation, degeneration and resorption of the associated articular facet, and the formation of a pseudoarthrosis between the bases of the first and second metatarsals (Fig S8). There is also a healed fracture of the right fifth metatarsal diaphysis.

There are no associated changes in the left talocrural, talocalcaneal or subtalar articulations or bones, all of which are normal. However, the left tibial and fibular diaphyses are posteriorly convex, rather than the usual straight to anteriorly convex tibial diaphyses (Fig S7F); although the tibia was reassembled from multiple pieces and that process may have produced some minor distortion, the associated fibular diaphysis is intact and matches the unusual curvature of the tibia. It is likely that this altered curvature, as well as the right talar trochlear flattening, are remodeling responses to altered stress patterns in the lower limb from a pathologically altered gait. The Shanidar 1 left tibial diaphysis, scaled to its length, was nonetheless as strong as those of other western Eurasian Neandertals [4].

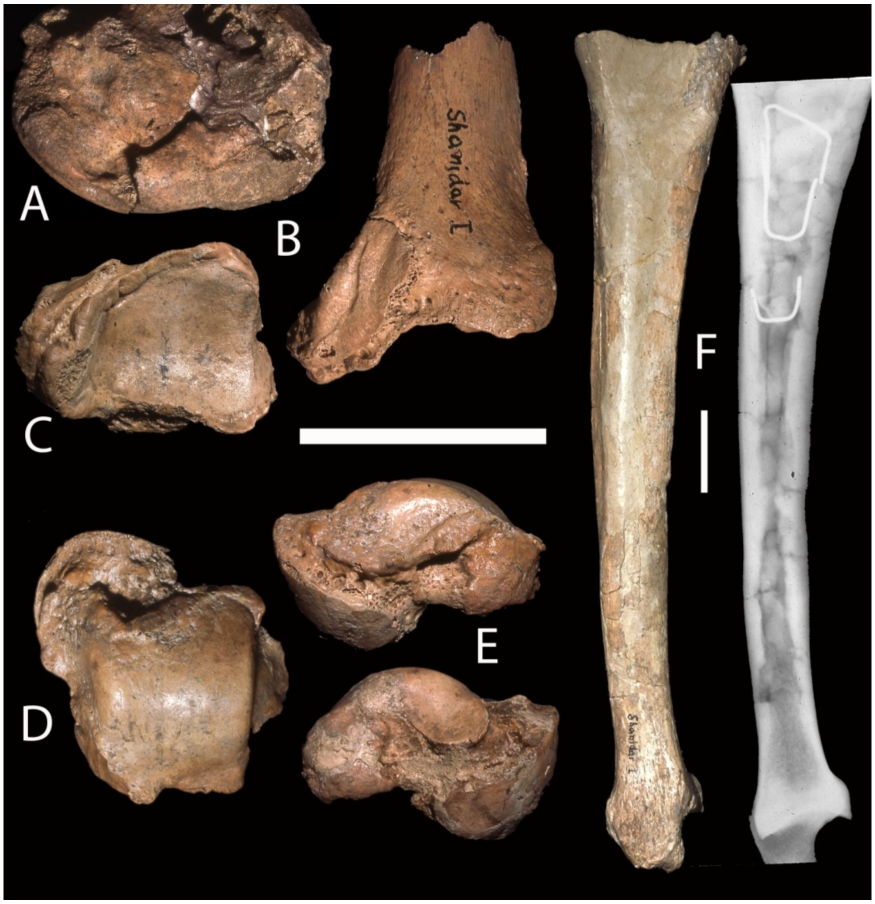

S7 Fig. **Abnormalities of the Shanidar 1 lower limbs**. A: periarticular exostoses on the lateral condylar surface of the right femoral condyle. B: posterior view of the distal right tibia, with ossification of the flexor hallucis longus bursa attachment. C: distal view of the right distal tibia, showing the new bone on the posterior malleolus and across the posterior trochlear articular margin. D: dorsal view of the right talus, with extra bony growths across the anterior trochlea and medial malleolar facet, plus on the medial posterior trochlea. E: medial views of the right (above) and left tali, comparing the dorsoplantarly flattened right talus with its talocrural articular lipping to the normal left talus. F: medial view of the left tibia and mediolateral radiograph of the tibia, with its pronounced anterior concavity; the curvature is not a product of reassembly given continuous contact across the original pieces and a matching posterior convexity in the associated left fibula. Scales: 5 cm.

Evidence for diffuse idiopathic skeletal hyperostosis

The Shanidar 1 remains exhibit a series of bony growths which were originally interpreted in terms of osteoarthritis [4] but subsequently recognized as reflecting diffuse idiopathic skeletal hyperostosis (DISH) [8]. DISH is a systemic disorder of unknown etiology that primary affects the thoracic and lumbar vertebrae and appendicular tendinous insertions [14]. It is particularly reflected in flowing, anterolateral, bridging ossifications between vertebral bodies, without involvement of the intervertebral disks or the articular facets. Secondarily it includes enthesopathies of major tendinous insertions, particularly those of triceps brachii,

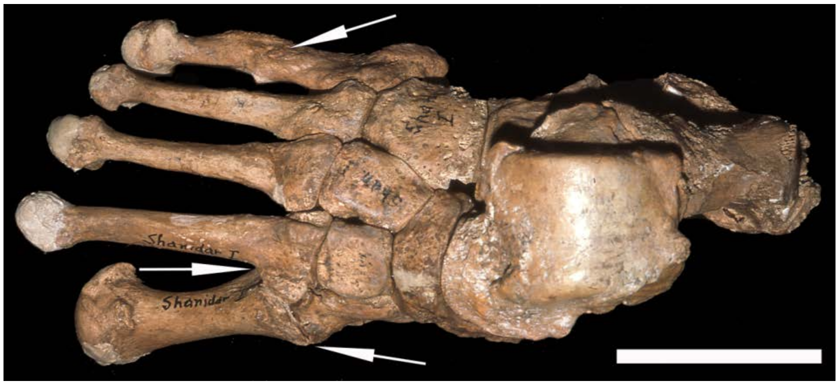

S8 Fig. **Dorsal view of the Shanidar 1 right tarsometatarsal skeleton**. The osteoarthritic changes of the first tarsometatarsal and proximal metatarsal 1-2 articulations are indicated, along with the healed fracture of the metatarsal 5 diaphysis. The talocrural alterations (Fig S7) and calcaneal enthesopathies (Fig S9) are evident, as well as a bony growth on the dorsal metatarsal 1 head. Scale: 5 cm.

quadriceps femoris, triceps surae and the plantar aponeurosis. Additional bony spurs may develop in the extremities.

The Shanidar 1 thoracic vertebrae are too fragmentary to assess for DISH, and the L1, L2 L4 and L5 bodies are crushed and/or missing their lateral portions. However, the largely complete L3 body (Fig S9A) has a prominent anterolateral flowing ossification, extending over the L3-L4 intervertebral space. The disk surfaces, although damaged, were not pathologically involved. Ideally, a diagnosis of DISH requires the presence of bridging ossifications across at least four vertebral bodies [14], but the fragmentary nature of the Shanidar 1 axial skeleton, especially the thoracic portion, makes it not possible to assess whether similar additional growths were present. There is a lumbar articular facet, probably of the L5, with an osteoarthritic enlargement, but none of the other disassociated lumbar and thoracic articular facets exhibit degenerations.

In the Shanidar 1 appendicular skeleton, the left proximal ulna has a triceps brachii enthesopathy on the olecranon (Fig S9B) (the right one was lost antemortem; see above). The patellae have growths for the quadriceps femoris tendons (Figs S9E and S9F), although the left ones are very small. On the calcanei, there are large and protruding insertion areas on the calcaneal tuberosities for the triceps surae (Achilles) tendons (Figs S9C and S9D), as well as distal extensions of the proximal attachments for the plantar aponeurosis.

Therefore, although the nature of the Shanidar 1 remains (incomplete vertebral column and the absence of various appendicular elements [4]) limits the diagnosis of DISH for this individual, all of these changes in concert strongly indicate the presence of this systemic abnormality. The form of the L3 growth in particular fits the pattern of DISH, as does the distribution of ulnar, patellar and calcaneal enthesopathies.

Shanidar 1 paleopathological implications

This suite of lesions on the Shanidar 1 remains indicates an individual who sustained serious trauma to the lateral face and right arm, secondary loss of the right forearm and hand, disruption of the right lower limb resulting in bilateral lower limb remodeling, and a systemic

degenerative condition. The orbital alteration may well have disrupted his left eye vision, especially if it affected the ocular muscles. The right shoulder and arm injury and eventual amputation (probably secondary to post-traumatic atrophy and non-union of the distal humeral fracture) left him with one functional upper limb and a stump for the other arm. The remodeling of the right talus and tibia indicate changed patterns of stress distribution in the legs and feet reflecting an abnormal gait. And the systemic DISH is normally associated with spinal and muscular stiffness producing reduced ranges of joint movement [14].

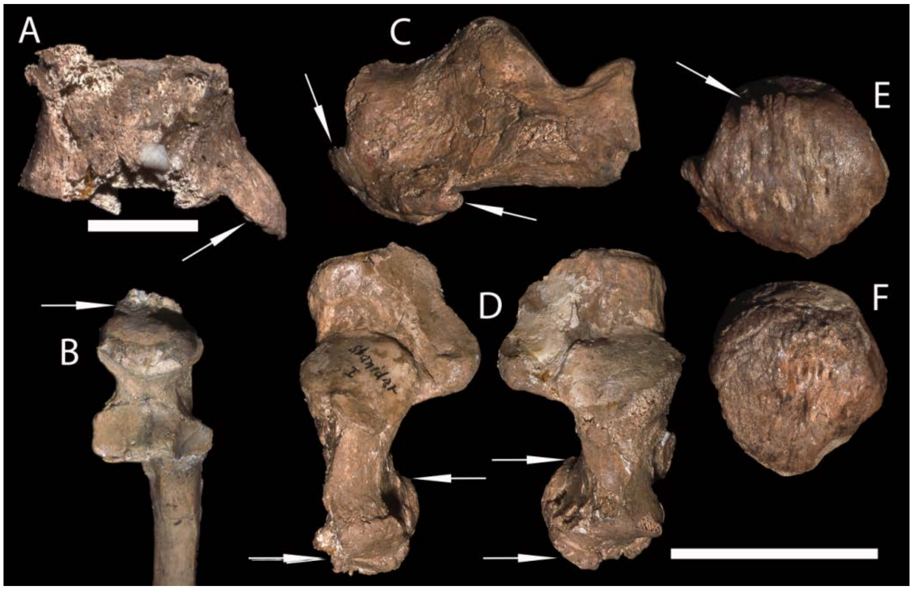

S9 Fig. **Ligamentous ossification and enthesopathies probably associated with diffuse idiopathic skeletal hyperostosis (DISH) on the Shanidar 1 skeletal remains**. A: ventral view of the L3 body, with a left anterolateral bridging ossification, but without involvement of the intervertebral disc surfaces. B: proximoanterior view of the left proximal ulna (the right one was absent at death – see above), with tendinous ossification of the triceps brachii tendon. C: lateral view of the right calcaneus with enthesopathies of the triceps surae (Achilles) tendon insertion and the lateral calcaneal origin of the plantar aponeurosis. D: dorsal views of the right and left calcanei, with enlargements of the triceps surae insertions and, especially on the right side, of the medial process for the plantar aponeurosis. E and F: right and left patellae in anterior view, with quadriceps femoris enthesopathies primarily on the right patella. L3 scale: 2 cm; scale for the other bones: 5 cm.

All of these conditions, except possibly the DISH, indicate an individual who sustained multiple insults, yet survived. More importantly, they indicate an individual whose normal functioning was compromised in several ways, including manipulation and burden carrying, locomotion and mobility on the landscape, and probably vision. And at least the right arm reduction extended through his more than two decades of mature life. As previously argued [4,6], long-term survival in this condition as a Middle Paleolithic forager in a Late Pleistocene environment without social support would have been extremely unlikely. His auditory exostoses and the implied conductive hearing loss only added to these impairments.

S1 References

1. Stewart TD. The restored Shanidar I skull. Sumer. 1958; 14(1):90–5.
2. Stewart TD. The Neanderthal skeletal remains from Shanidar Cave, Iraq: A summary of findings to date. Proc Am Phil Soc. 1977; 121(2):121-65.
3. Solecki RS. Three adult Neanderthal skeletons from Shanidar Cave, northern Iraq. Annu Rep Smithsonian Inst. 1960; 1959:603-35.
4. Trinkaus E. The Shanidar Neandertals. New York: Academic; 1983.
5. Trinkaus E, Zimmerman MR. Trauma among the Shanidar Neandertals. Am J Phys Anthropol. 1982; 57(1):61-76.
6. Stewart TD. Fossil evidence for human violence. Trans-Action. 1969; 6(7):48-53.
7. Lietava J. A differential diagnostics of the right shoulder girdle deformity in the Shanidar I Neanderthal. Anthropol (Brno). 1988; 26(3):183-96.
8. Crubézy E, Trinkaus E. Shanidar 1: A case of hyperostotic disease (DISH) in the Middle Paleolithic. Am J Phys Anthropol. 1992; 89(4):411-20.
9. Wu XJ, Schepartz LA, Liu W, Trinkaus E. Antemortem trauma and survival in the Late Middle Pleistocene human cranium from Maba, south China. Proc Natl Acad Sci USA. 2011; 108(49):19558-62.
10. Martín-Francés L. Revisión y Estudio des las Manifestaciones Paleopatológicas en los Homininos del Plio-Pleistoceno, con Especial Referencia a Algunos Fósiles de la Sierra de Atapuerca [dissertation]. Alcalá: Universidad de Alcalá; 2015.
11. Trinkaus E, Churchill SE. Diaphyseal cross-sectional geometry of Near Eastern Middle Paleolithic humans: The humerus. J Archaeol Sci. 1999; 26(2):173-84.
12. Stewart TD. Nonunion of fractures in antiquity, with description of five cases from the New World involving the forearm. Bull NY Acad Med. 1974; 50(8):875-91.
13. Trinkaus E, Thompson DD. Femoral diaphyseal histomorphometric age determinations for the Shanidar 3, 4, 5 and 6 Neandertals and Neandertal longevity. Am J Phys Anthropol. 1987; 72:123-29.
14. Resnick D. Diffuse idiopathic skeletal hyperostosis. In: Resnick D, editor. Diagnosis of Bone and Joint Disorders, 4th ed. Philadelphia: Saunders; 2002; 2:1476-503.
