# Supplementary material for: External auditory exostoses and hearing loss in the Shanidar 1 Neandertal
Source: PLoS One. 2017 Oct 20;12(10):e0186684. doi: 10.1371/journal.pone.0186684 (PMC5650169; doi:10.1371/journal.pone.0186684)
Supplement: S2 Text — (PDF) [file pone.0186684.s002.pdf]

External Auditory Exostoses and Hearing Loss in the Shanidar 1 Neandertal Supporting Information Appendices

Erik Trinkaus[1] and Sébastien Villotte[2]

[a] Department of Anthropology, Washington University, Saint Louis MO 63130, USA
[b] UMR5199 PACEA, Université de Bordeaux – CNRS, Bâtiment B8, Allée Geoffroy Saint Hilaire, CS 50023, 33615 Pessac cedex, France

S2 Text: Neandertal external auditory exostoses

In addition to Shanidar 1, at least four Neandertals exhibit external auditory exostoses (EAE) that are relatively large, although less pronounced than those of Shanidar 1. Their EAE are best scored as Grade 2 (see Methods). For visual comparisons, lateral views of them are provided here. The EAE of La Chapelle-aux-Saints 1 and Krapina 39.1 have been mentioned and/or illustrated previously [1-5]; to our knowledge the Spy 1 and Tabun 1 EAE have not been previously described.

La Chapelle-aux-Saints 1 is 35-45 year old male based primarily on iliac auricular metamorphosis and secondarily on the wear of the remaining premolars despite his extensive antemortem tooth loss [1]. Its EAE consist primarily of two on each side. In the right auditory canal, there is a small posterior one extending mostly laterally and a much larger anterior one extending posteriorly into the canal laterally (Figs S10 and S11). The left meatus has an exostosis anteriorly that is mostly in the lateral portion of the canal and then a larger one posteriorly that is placed more medially within the canal.

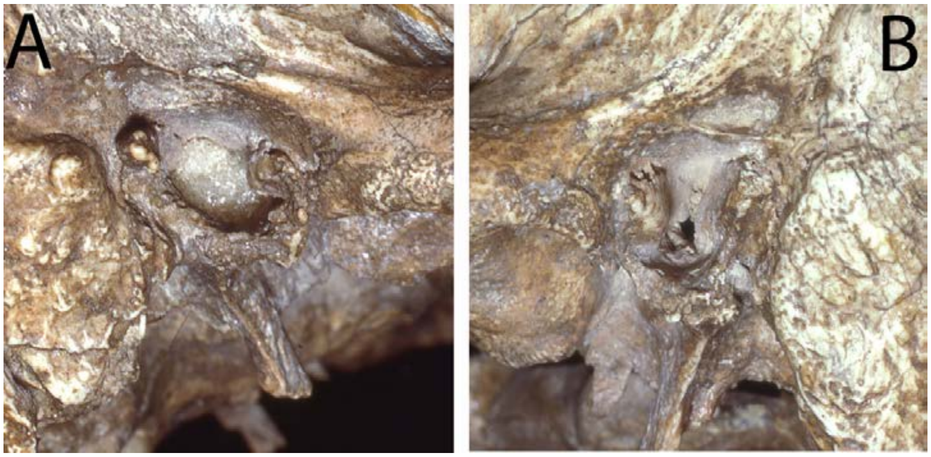

S10 Fig. Lateral views of the La Chapelle-aux-Saints 1 right (A) and left (B) auditory meatus.

Spy 1 is a young adult partial skeleton, its age based on the moderate dental occlusal wear. Its right auditory canal (Fig S12A) has a largely normal anterior porus and inferior tympanic region. However, the posterior portion of the tympanic bone onto the posterosuperior

meatus possesses two large exostoses, one primarily posteroinferior and the other posterosuperior. The more superior one is evenly rounded laterally, but the inferior one has an irregular lateral portion and a more protruding swelling that extends into the canal.

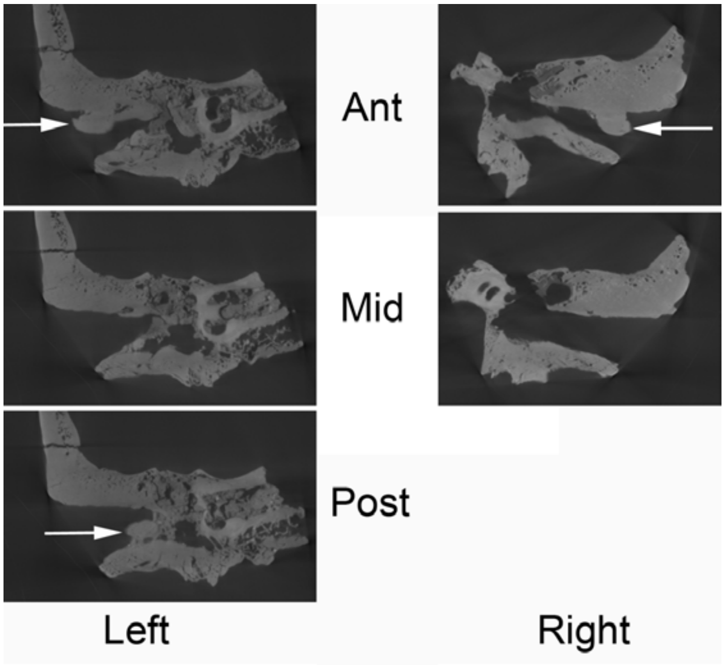

S11 Fig. **Coronal CT slices through the La Chapelle-aux-Saints 1 left and right temporal bones** [CT images at 90 µm provided by A. Balzeau and freely available for research through the Musée de l'Homme (http://colhelper.mnhn.fr/)]. The "Mid" slices are through the unencumbered middles of the meatus; the left one contains sediment. The "Ant" slices are through the maximum developments of the right and left anterior exostoses. The left "Post" slice is through the more medial posterior exostosis. The right posterior exostosis is primarily lateral at the porus (Fig S10), and it is not evident in the coronal CT slices. The exostoses are indicated by the arrows.

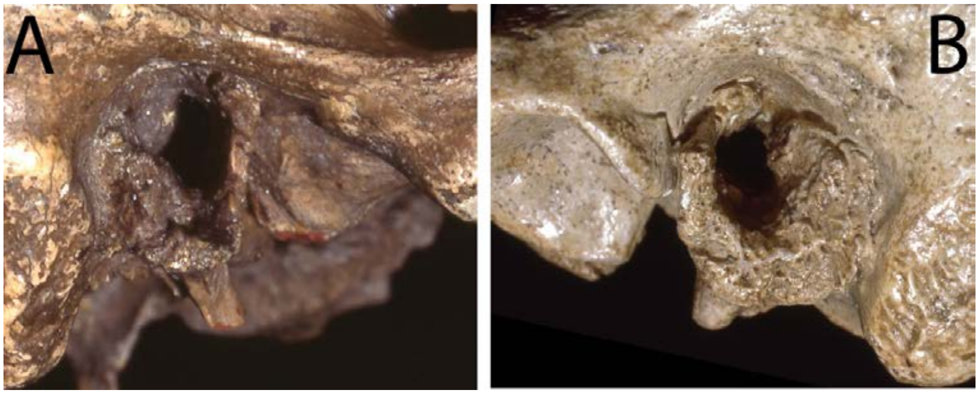

S12 Fig. **Lateral views of the Spy 1 right auditory canal (A) and the Krapina 39.1 left auditory canal (B).**

Krapina 39.1 is an isolated temporal bone; it is fully mature, and the generally young age-at-death and absence of older adults in the large Krapina sample (2) suggests that it derives from a young adult. The anterior and inferior portions of the lateral tympanic bone are thickened, and that thickness becomes exaggerated in the posterior tympanic bone (Fig S12B). However, the posteroinferior thickening is exaggerated by irregular bone extending into the auditory canal as a convex but irregular swelling. Superior to the swelling is a more discrete exostosis along the posterosuperior lateral canal. These bony growth are accompanied by a small one just anterior of the superior apex of the canal.

Tabun 1 is a younger adult female partial skeleton, age and sex based on dental wear and pelvic morphology respectively [6]. Her external auditory canals (Fig S13) are poorly preserved and the tympanic bones are largely absent inferiorly. The presence of an exostosis in the left meatus is unclear, given fossilization damage. The right one, however, has a large swelling in the posteroinferior canal along the tympanic bone, and there is a broad exostosis anterosuperiorly extending medially into the meatus. The two bony growths, especially more medially than the porus, converge to produce a marked narrowing of the canal posterosuperiorly.

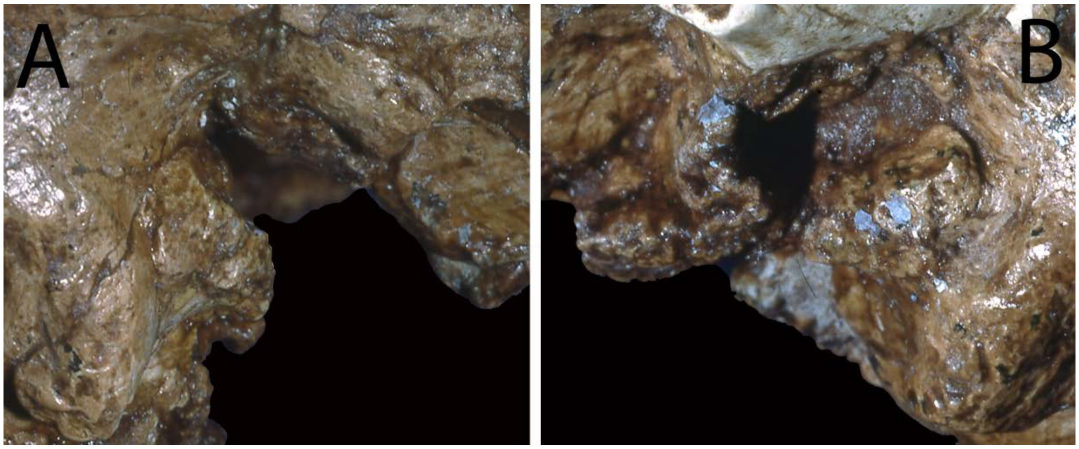

S13 Fig. **Lateral views of the Tabun right (A) and left (B) auditory canals and adjacent bone**. Both meatus are missing most of the inferior tympanic bone, and the left one exhibits further erosion.

Despite the variations in the forms and positions of these exostoses, each of these Neandertals exhibits marked narrowing of the meatus on at least one side. Each one is scored Grade 2 as a result.

S2 References

1. Trinkaus E. Pathology and the posture of the La Chapelle-aux-Saints Neandertal. Am J Phys Anthropol. 1985; 67(1):19-41.
2. Radovčić J, Smith FH, Trinkaus E, Wolpoff MH. The Krapina Hominids: An Illustrated Catalog of the Skeletal Collection. Zagreb: Mladost; 1988.
3. Boule M. L'homme fossile de La Chapelle-aux-Saints. Ann Paléontol. 1911-13; 6:111-72; 7:21-56, 85-192; 8:1-70.
4. Heim JL. Les hommes fossiles de La Ferrassie I: Le gisement. Les squelettes adultes (crâne et squelette du tronc). Arch Inst Paléontol Hum. 1976; 35:1-331.

5. Condemi S. Les Néandertaliens de La Chaise. Paris: Comité des Travaux Historiques et Scientifiques; 2001.
6. McCown TD, Keith A. The Stone Age of Mount Carmel II: The Fossil Human Remains from the Levalloiso-Mousterian. Oxford: Clarendon; 1939.
