# Supplementary material for: External auditory exostoses and hearing loss in the Shanidar 1 Neandertal
Source: PLoS One. 2017 Oct 20;12(10):e0186684. doi: 10.1371/journal.pone.0186684 (PMC5650169; doi:10.1371/journal.pone.0186684)
Supplement: S3 Text — (PDF) [file pone.0186684.s003.pdf]

External Auditory Exostoses and Hearing Loss in the Shanidar 1 Neandertal Supporting Information Appendices

Erik Trinkaus[1] and Sébastien Villotte[2]

[a] Department of Anthropology, Washington University, Saint Louis MO 63130, USA

[b] UMR5199 PACEA, Université de Bordeaux – CNRS, Bâtiment B8, Allée Geoffroy Saint Hilaire, CS 50023, 33615 Pessac cedex, France

S3 Text: Differential diagnosis

The differential diagnosis for EAE includes osteomata. These sutural protrusions are pedunculated bone formations, usually solitary and unilateral [1,2]. Figure S14 illustrates probable cases of osteomata (black arrow) along the tympanosquamous suture, associated with a modest external auditory exostosis (white arrow) on the anteroinferior tympanic bone. The probable osteomata were mistakenly presented by Villotte and Knüsel [3] as external auditory exostoses.

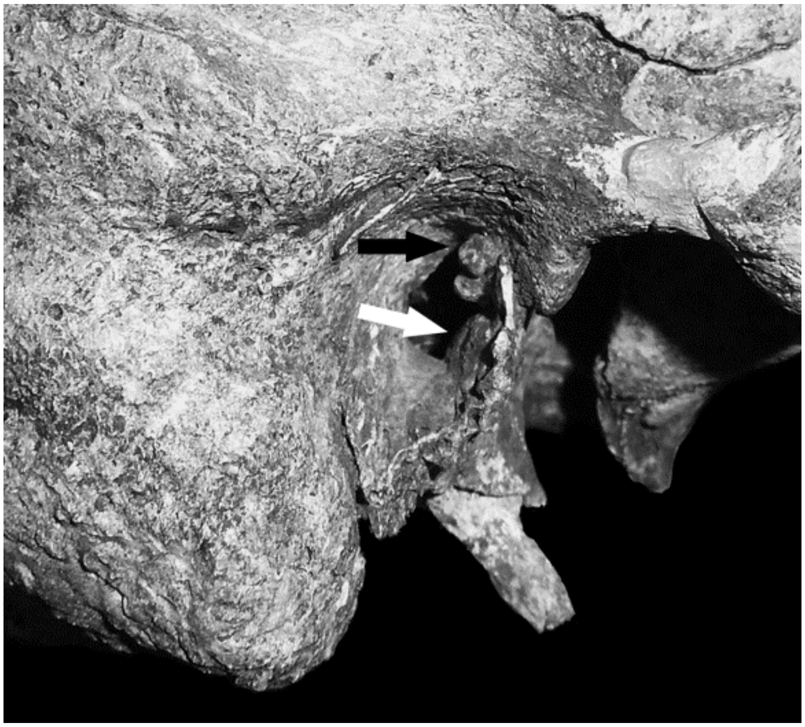

S14 Fig. Recent human right temporal bone with osteomata (black arrow) and an external auditory exostosis (white arrow).

S3 References

1. Graham MD. Osteomas and exostoses of the external auditory canal. A clinical, histopathologic and scanning electron microscopic study. Ann Otol Rhinol Laryngol. 1979; 88: 566–72.
2. Leonetti JP, Marzo SJ. Diseases of the external auditory canal. In: Pensak ML, Choo DI, editors. Clinical Otology, 4th ed. New York: Thieme; 2015. p. 181-91.
3. Villotte S, Knüsel CJ. External auditory exostoses and prehistoric aquatic resource procurement. J Archaeol Sci Rep. 2016; 6(4):633-6.
